# A Quantitative ELISA to Detect Anti-SARS-CoV-2 Spike IgG Antibodies in Infected Patients and Vaccinated Individuals

**DOI:** 10.3390/microorganisms10091812

**Published:** 2022-09-09

**Authors:** Ji Luo, Jennifer Klett, Jörg Gabert, Thomas Lipp, Julia Karbach, Elke Jäger, Stephan Borte, Ralf Hoffmann, Sanja Milkovska-Stamenova

**Affiliations:** 1 Institute of Bioanalytical Chemistry, Faculty of Chemistry and Mineralogy, Universität Leipzig, Deutscher Platz 5, 04103 Leipzig, Germany; 2 Center for Biotechnology and Biomedicine, Universität Leipzig, Deutscher Platz 5, 04103 Leipzig, Germany; 3 Adversis Pharma GmbH, Deutscher Platz 5, 04103 Leipzig, Germany; 4 Gemeinschaftspraxis Lipp/Amm/Lipp, Karl-Liebknecht-Straße 103, 04275 Leipzig, Germany; 5 Klinik für Onkologie und Hämatologie, Krankenhaus Nordwest, Steinbacher Hohl 2, 60488 Frankfurt, Germany; 6 Department of Laboratory Medicine, Hospital St. Georg gGmbH, Delitzscher Str. 141, 04129 Leipzig, Germany; 7 ImmunoDeficiencyCenter Leipzig, Jeffrey Modell Diagnostic and Research Center for Primary Immunodeficiency Diseases, Hospital St. Georg gGmbH, Delitzscher Str. 141, 04129 Leipzig, Germany

**Keywords:** COVID-19, SARS-CoV-2, spike protein, vaccination, serological test, ELISA

## Abstract

There is an ongoing need for high-precision serological assays for the quantitation of anti-SARS-CoV-2 antibodies. Here, a trimeric SARS-CoV-2 spike (S) protein was used to develop an ELISA to quantify specific IgG antibodies present in serum, plasma, and dried blood spots (DBS) collected from infected patients or vaccine recipients. The quantitative S-ELISA was calibrated with international anti-SARS-CoV-2 immunoglobulin standards to provide test results in binding antibody units per mL (BAU/mL). The assay showed excellent linearity, precision, and accuracy. A sensitivity of 100% was shown for samples collected from 54 patients with confirmed SARS-CoV-2 infection more than 14 days after symptom onset or disease confirmation by RT-PCR and 58 vaccine recipients more than 14 days after vaccination. The assay specificity was 98.3%. Furthermore, antibody responses were measured in follow-up samples from vaccine recipients and infected patients. Most mRNA vaccine recipients had a similar response, with antibody generation starting 2–3 weeks after the first vaccination and maintaining positive for at least six months after a second vaccination. For most infected patients, the antibody titers increased during the second week after PCR confirmation. This S-ELISA can be used to quantify the seroprevalence of SARS-CoV-2 in the population exposed to the virus or vaccinated.

## 1. Introduction

Since December 2019, the novel severe acute respiratory syndrome coronavirus 2 (SARS-CoV-2) has spread worldwide, leading to a global pandemic. According to the latest WHO report, over 574 million confirmed SARS-CoV-2 infections, and over 6.3 million deaths have been reported globally [1]. It is likely that there are many more undiagnosed cases with mild or no symptoms.

SARS-CoV-2 is an enveloped, single-stranded RNA virus belonging to the genus *Betacoronavirus* and the species *Severe acute respiratory syndrome-related coronavirus* [2]. The similarities between severe acute respiratory syndrome coronavirus (SARS-CoV-1), Middle East respiratory syndrome coronavirus (MERS-CoV), and SARS-CoV-2 have been investigated in the light of available data [3]. SARS-CoV-2 is by far more infectious than SARS-CoV-1 and MERS-CoV, but has a lower lethality rate [4,5]. Although the US Food and Drug Administration (FDA) has approved one drug treatment for COVID-19 (antiviral drug Veklury (remdesivir)) and has authorized others for emergency use [6], there is still no recommended global treatment available. The approach to containing SARS-CoV-2 spreading is still based on preventing infections through controlling social distance, wearing masks, and vaccination. There is now solid evidence that establishing physical prophylaxis, such as lockdowns, social distancing, extensive wearing masks, was only partially effective, but instead had a negative impact on society and economic development [7,8,9]. Therefore, universal vaccination appears to be the most important tool to avoid heavy symptoms and deaths from SARS-CoV-2 infection. In response to the major public health crisis of COVID-19, there is a global effort to develop different SARS-CoV-2 vaccines using various technologies [10,11,12]. Currently, there are three main types of effective vaccines produced by Pfizer-BioNTech (mRNA), Moderna (mRNA), and Oxford/AstraZeneca (chimpanzee adenovirus vector ChAdOx-1) on the European market [13].

Most available vaccines target the spike (S) protein of the virus due to its role in cell binding and entry [14,15]. The S protein is a trimeric protein comprising two subunits, namely S1 and S2 [16]. The S1 subunit mediates binding to host cells via interactions between its receptor-binding domain (RBD) and the human receptor angiotensin-converting enzyme 2 (ACE2), whereas the S2 subunit is responsible for membrane fusion, which is required for virus entry [17,18]. Although the nucleocapsid (N) protein is also highly immunogenic and a major target for antibody response, it has not been considered for vaccine production due to earlier studies showing that vaccines expressing this protein did not provide protection, but also caused infection-induced pneumonia [19,20]. The other viral proteins (membrane, M and envelope, E) have not been studied as vaccine targets due to their poor immunogenicity [21].

Currently, about 67% of the world population has received at least one dose of a COVID-19 vaccine, and over 62% population has been vaccinated completely [22]. With the spread of the COVID-19 pandemic, it is foreseeable that vaccination against SARS-CoV-2 will be the largest vaccination program of the century. Usually, vaccination performs its protective role by stimulating humoral or cellular immune response in the host, but the individual humoral response to COVID-19 remains largely unpredictable. It has been reported that antibody titers measured in enzyme-linked immunosorbent assays (ELISA) against the spike protein correlate with virus neutralization [23]. Thus, quantitation of anti-S antibodies may allow following and understanding of the immune response after vaccination and establishing correlations of protection against SARS-CoV-2. Anti-S antibody assays may also provide a better retrospective assessment of infections, since they might be more specific than anti-N antibodies, which may be more sensitive in early infection phases.

Indirect enzyme-linked immunosorbent assay (iELISA) is a suitable biochemical tool that is commonly used for specific antibody detection. Over the past two years, a large number of antibody tests with different performance characteristics have emerged [24]. However, most of them only provide results defining infected/uninfected or effective/ineffective vaccination; few tests can provide quantitative results, and most provide results based only on antibody concentration (ng/mL) [25]. These tests do not effectively reflect the changes in antibody value in the body after infection and do not allow for a proper assessment of vaccination efficiency.

In this study, we report an iELISA for quantitative detection of SARS-CoV-2-specific IgG antibodies against the spike protein. This assay was calibrated with international anti-SARS-CoV-2 immunoglobulin standards (i.e., the WHO standard, which enables reporting test results in binding antibody units per mL; BAU/mL) and can be used with various sample matrices, including serum, plasma, and dried blood spots (DBS). The linearity, accuracy, precision, sensitivity, specificity, and cut-off values of the assay were validated using two WHO International Standards for anti-SARS-CoV-2 immunoglobulin, 54 sera collected from persons with PCR-confirmed SARS-CoV-2 infections, 58 serum or plasma from vaccinated persons, and 173 control samples collected before 2019.

## 2. Materials and Methods

### 2.1. Reagents

Reagents were obtained from the following manufacturers: BBI Solution (Crumlin, UK): IgG depleted serum; Biomat (Trentino, Italy): High Binding 96-well strip plates; Brand (Werheim, Germany): 96-well strip plate (12 × F8), medium binding; Carl Roth GmbH & Co. KG (Karlsruhe, Germany): ROTI^®^Fair carbonate bicarbonate buffer pH 9.6, ROTI^®^Stock 10× PBS, ROTI^®^Stock 10× PBS-T and sulfuric acid (0.5 M); ExcellGene SA (Monthey, Valais, Swiss): trimeric spike protein of SARS-CoV-2 (1.3 mg/mL); GenScript Biotech BV (Leiden, The Netherlands): SARS-CoV-2 spike protein mAbs; MORPHISTO GmbH: acetate buffer pH 5.0; Greiner Bio-One (Frickhausen, Germany): Microplate, 96-well, PS, F-bottom, clear; Indical Bioscience (Leipzig, Germany): sample buffer; PAN-Biotech GmbH (Aidenbach, Germany): human serum (sterile filtered); PerkinElmer Health Sciences, Inc. (Greenville, SC, USA): 226 Spot Saver RUO Card, CE-certified; National Institute for Biological Standards and Control (Potters Bar, Hertfordshire, UK): First WHO International Standard for anti-SARS-CoV-2 immunoglobulin (human) NIBSC code: 20/162 working reagent for anti-SARS-CoV-2 immunoglobulin NIBSC code: 21/234; Promega GmbH (Mannheim, Germany): peroxidase-conjugated anti-human IgG antibody; Seramun Diagnostika GmbH (Heidesee, Germany): 3,3’,5,5’-tetramethylbenzidine (TMB) peroxidase substrate solution; SERVA Electrophoresis GmbH (Heidelberg, Germany): acrylamide/bis(acrylamide); Surmodics IVD, Inc. (Eden Prairie, MN, USA): StabilZyme^TM^ SELECT; Thermo Fisher Scientific (Waltham, MA, USA): AcroMetrix™ Inhibition Panel; SuperBlock^TM^ blocking buffer in PBS; F8 Maxisorp Nunc-Immuno Module.

### 2.2. Sample Collection

Serum, plasma, and/or DBS samples were collected from AstraZeneca, Pfizer-BioNTech and Moderna COVID-19 vaccine recipients (Gemeinschaftspraxis Lipp/Amm/Lipp, Leipzig, Germany; Supplementary Table S1) and Hospital St. Georg Leipzig (Leipzig, Germany; Supplementary Table S3). DBS samples (*n* = 128) of Moderna, Pfizer-BioNTech, AstraZeneca, and Johnson & Johnson vaccine recipients were obtained from 25 independent donors (Supplementary Table S2). Serum samples from PCR-confirmed SARS-CoV-2 positive patients were obtained from hospitalized patients (Krankenhaus Nordwest, Frankfurt, Germany, and Hospital St. Georg gGmbH, Leipzig, Germany; Supplementary Tables S4 and S5). Control sera collected between 2012 and 2018 were enriched for older people and those with a history of cancer or rheumatoid arthritis to specifically test the assay specificity in populations at higher risk for severe COVID-19 conditions and with presumably a higher incidence of previous viral infections, including other coronaviruses, although this information was not available (Supplementary Table S6). These 173 serum samples were obtained from the Krankenhaus Nordwest (Frankfurt, Germany), from patients with rheumatoid arthritis (Gemeinschaftspraxis für Internistische Rheumatologie, Leipzig, Germany) as well as from independent donors. Furthermore, serum samples from persons antibody-positive for other common viral diseases (human immunodeficiency viruses (HIV) 1/2, hepatitis A/B virus, cytomegalovirus, Epstein–Barr virus, herpes simplex virus, human coronavirus (hCoV) HKU-1, influenza A/B) were used for cross-reactivity studies (INSTAND, Düsseldorf, Germany).

### 2.3. Dry Blood Spot Elution Procedure

DBS samples were collected from a fingertip using the AProof^®^ Sampling Set (Adversis Pharma GmbH, Leipzig, Germany) following the manufacturer’s instructions and stored at 20 °C. Prior to analysis, one single DBS punch with a diameter of 4.7 mm was incubated in sample buffer (Indical Bioscience, Leipzig, Germany, 250 µL, 37 °C, 1 h) for antibody elution.

### 2.4. Indirect ELISA

An indirect ELISA for IgG antibodies specific to the SARS-CoV-2 S protein in serum, plasma, and dried blood spots was developed. Nunc MaxiSorp™ 96-well microplates were coated with 200 ng/well of recombinant trimeric S protein and incubated at 4 °C overnight. Plates were washed three times with PBS and 1% Tween-20 (PBS-T) and then blocked with SuperBlock^TM^ buffer (200 µL/well room temperature (RT), 30 min). The blocking buffer was then discarded. Serum and plasma samples were diluted at 1:100 with sample buffer (Indical Bioscience, Leipzig, Germany), 100 µL of diluted samples or DBS eluates were transferred into the corresponding wells and incubated at RT for 45 min. After washing three times with PBS-T, 100 µL of conjugate solution was added (anti-Human IgG-HRP, 1:50,000 in StabilZyme^TM^ SELECT) and incubated at RT for 30 min. Plates were again washed three times as described above, and 100 µL of TMB substrate solution was added to each well. The reaction was stopped after 10 min by adding 100 µL of sulfuric acid (0.3 mol/L), and the absorbance was recorded at 450 nm using a SUNRISE microplate reader (Tecan Group AG, Männedorf, Switzerland). Human serum (off-the-clot, sterile filtered; PAN-Biotech GmbH, Aidenbach, Germany) was used as a negative control. A pooled plasma sample NIBSC 21/234 from WHO International Laboratory for Biological Standards was used as a positive control. The calibration curve was generated by a serial dilution of an anti-S1 antibody (Genscript Biotech BC, Leiden, Netherlands) in IgG depleted serum (BBI Solution, Crumlin, UK) to achieve the following values 428.2, 258.4, 142.7, 71.4, 35.7, and 3.6 BAU/mL. The limit of detection (LOD) was based on the standard deviation (SD) of the response and slope of the dilution curve: LOD = 3.3 σ/S. The limit of quantitation was determined based on the SD of the slope: LOQ = 10 σ/S.

To test whether interfering substances affect the ELISA, a positive serum sample was 128-fold diluted with plasma containing different interfering substances, including hemoglobin (up to 20 mg/mL), bilirubin (up to 0.3 mg/mL), triglycerides (up to 15 mg/mL), and heparin (up to 3000 U/L). Cross-reactivity was determined using 29 serum samples which were antibody positive for various other diseases (HIV 1/2, parvovirus B19, hepatitis A/B virus, cytomegalovirus, Epstein–Barr virus, herpes simplex virus, hCoV HKU-1, influenza H1N1, and influenza H5N1) collected before the COVID-19 pandemic started in 2019. Stability of ELISA was tested using coated ELISA plates and all materials for testing stored at 37 °C for accelerated stability test for nine time points over 42 days. Data analysis was performed using Microsoft Excel 2019 (Microsoft Office Professional Plus 2019) and GraphPad Prism 9.0.2 (Graph Pad Software, La Jolla, CA, USA).

## 3. Results

### 3.1. Development of SARS-CoV-2 Spike Protein-Specific Quantitative IgG ELISA

An indirect ELISA for anti-SARS-CoV-2 spike IgG antibodies was established using a recombinant trimeric spike protein. To optimize the coating conditions, four microtiter plate types including maxisorp (Nunc), which consists of a modified, highly charged polystyrene surface with high affinity to molecules with polar or hydrophilic groups, medium binding (Brand; Greiner), and high binding (Biomat) were compared using a positive serum pool and the negative serum pool (Supplementary Figure S1A). Among them, the maxisorp plate and the medium binding plate from Brand provided the best signal-to-noise (S/N) ratio. The maxisorp plate was preferred for all further experiments, as it is routinely used for other assays in our laboratory. Three coating buffers, i.e., carbonate buffer (pH 9.6), PBS (pH 7.4), and acetate buffer (pH 5.0), were compared using a serum pool collected from infected patients and a pool of negative sera (Supplementary Figure S1B). PBS and acetate buffer showed comparable S/N ratios between positive and negative pools, and both buffers were superior to carbonate buffer. PBS was chosen as the coating buffer, as it is commonly used in our standard procedures. When trimeric S protein was coated in PBS at quantities from 50 ng to 300 ng per well, the OD450 values increased from 2.809 to 3.836. As the plate was saturated with 200 ng per well in PBS (Supplementary Figure S1C), all following experiments relied on these coating conditions and a 50,000-fold dilution of the secondary anti-Human IgG-HRP antibody in Stabilzyme Select, which appeared to have a higher S/N ratio than other tested dilutions (Supplementary Figure S1D).

The antibody levels in human samples were quantified using a calibration curve generated by a serial dilution of an anti-S1 antibody in an IgG depleted serum to achieve the values of 428.2, 258.4, 142.7, 71.4, 35.7, and 3.6 BAU/mL. The antibody levels of the calibration standards were converted into BAU/mL and validated using a WHO International Standard for anti-SARS-CoV-2 immunoglobulin (NIBSC 20/162) and a pooled plasma sample (NIBSC 21/234) from the WHO International Laboratory for Biological Standards. NIBSC 21/234 and a human serum (off-the-clot, sterile filtered) were used as positive and negative controls on each plate, respectively.

### 3.2. Linearity

In order to verify that sample matrices containing the detection analyte provide reliable quantitation after dilution within the standard curve range, dilution series of the NIBSC 20/162 (1:125 to 1:8,192,000) were analyzed as described before. Excellent linearity was achieved in the range of 464.4 to 0.031 BAU/mL (dilutions 1:1000 to 1:8,192,000) with coefficients of variation below 10% (Table 1 and Supplementary Figure S2) defining the measurement range. The upper quantification limit is 428.2 BAU/mL. To determine antibody levels above the upper limit of quantification, the samples should be analyzed after dilution.

### 3.3. Precision and Reproducibility

Precision and reproducibility of the anti-S-IgG ELISA were evaluated using a positive pool (generated by pooling sera from eight probands collected more than two weeks after receiving a second Pfizer-BioNTech or Moderna vaccine) by using five positive pool dilutions ranging from 1:1000 to 1:10,000 covering the measurement range of the calibration curve. The analyses were performed by different operators on different days and using different batches. Each pool dilution was additionally tested in triplicate, and the coefficient of variation (CV) was used to evaluate the precision of the assay. The intra-assay, inter-operator, inter-day, and inter-batch CVs were all lower than 10% (Table 1 and Supplementary Table S8).

### 3.4. Accuracy

**Figure 1:**
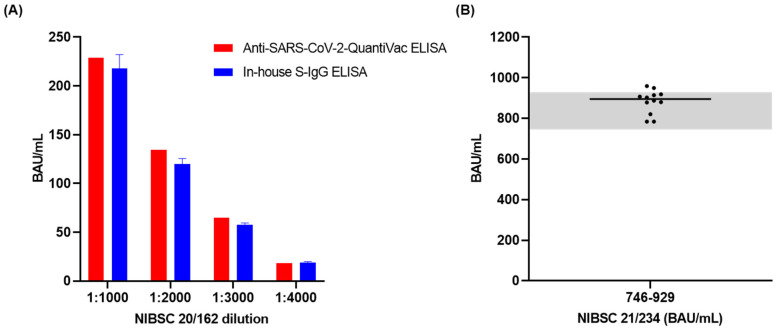
Accuracy of in-house SARS-CoV-2 S-IgG ELISA (blue) determined (**A**) in comparison to Euroimmun, Anti-SARS-CoV-2-QuantiVac-ELISA (red) using NIBSC 20/162 in five replicates and four dilutions (1:1000, 1:2000, 1:3000, 1:4000); standard deviations are shown as error bars (blue) and (**B**) using a standard with known concentration (NIBSC 21/234, 832 BAU/mL) analyzed in twelve replicates on four different days. The grey area indicates the anti-S titer of NIBSC 21/234 of 746–929 BAU/mL (95% CI).

The accuracy of the developed assay was determined in a comparison with commercial ELISA and using a standard of known antibody levels. The NIBSC standard 20/162 with an unknown level of anti-S antibodies was analyzed in four different dilutions, each in five replicates, by our in-house assay and a commercial ELISA (Euroimmun, Anti-SARS-CoV-2-QuantiVac-ELISA). The recoveries calculated based on the antibody levels determined by the Euroimmun ELISA ranged from 89.3 to 102% with CVs below 7% (Figure 1A). Furthermore, the standard 21/234, which has a defined an anti-S antibody level of 832 BAU/mL (95% CI: 746–929 BAU/mL), was measured in twelve replicates on four different days, showing recoveries of 106% with CV below 7% (Figure 1B), indicating very good accuracy.

### 3.5. Analytical Sensitivity

The analytical sensitivity of in-house SARS-CoV-2 anti-S-IgG ELISA was determined using the WHO standard NIBSC 20/162 in 10 dilutions (1:16,000 to 1:8,192,000 each in eight replicates and the analysis was performed on three different plates. LOD and LOQ were 0.114 BAU/mL and 0.345 BAU/mL, respectively (Table 1 and Supplementary Table S7).

### 3.6. Diagnostic Sensitivity and Specificity of the S-IgG ELISA

**Figure 2:**
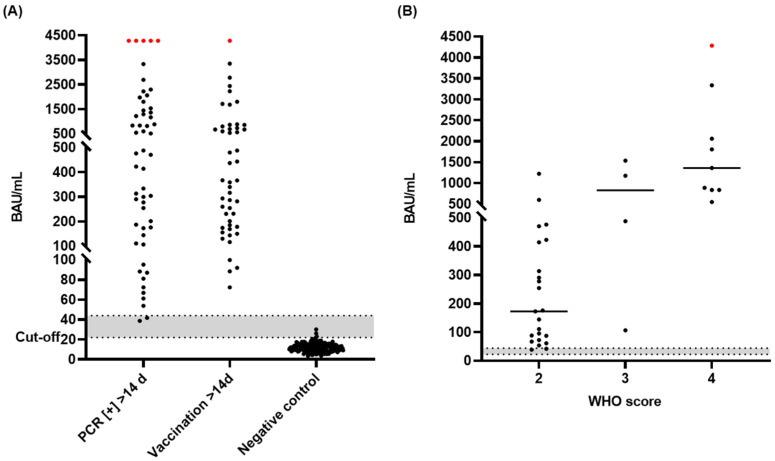
Anti-S antibody titers (BAU/mL) of samples from SARS-CoV-2-infected patients, COVID-19 vaccine recipients, and negative controls. (**A**) A total of 54 serum samples from persons infected with SARS-CoV-2 collected at least 14 days after a positive PCR-test, 58 samples collected from vaccinated donors 14 days or longer after first vaccine (Pfizer-BioNTech, Moderna, and AstraZeneca), and 173 serum samples collected before 2018. (**B**) Serum samples (36) collected from persons infected with SARS-CoV-2 (>14 days after positive PCR test) were grouped by severity of symptoms based on their WHO score (2 to 4). Results were converted to binding antibody units (BAU/mL) using the absorbances recorded at 450 nm of six calibrators. The highest positive (red) have a titer of >4280 BAU/mL because the analysis was performed up to this value. The cut-off was 22 BAU/mL for the in-house ELISA. The grey zone above the cut-off (22–44 BAU/mL) indicates a borderline range.

The SARS-CoV-2 spike-protein-specific IgG quantitative ELISA was validated using samples from infected patients, vaccine recipients, and healthy controls. Antibody titers were determined for 54 serum samples collected from hospitalized infected patients at least 14 days after symptoms onset or disease confirmation by RT-PCR, 58 serum or plasma samples collected from donors around 14 days after vaccination, and 173 pre-pandemic serum samples as control sera indicating a good separation between positive and negative samples (Figure 2A). Based on this experiment, the assay cut-off calculated as the mean of the antibody levels of the negative sera plus three standard deviations was defined as 22 BAU/mL. Among the negative serum samples, three samples tested false positive, indicating a specificity rate of 98.3% (95% CI, 95.0% to 99.6%). Values between 22 and 44 BAU/mL were considered borderline as they fell in the very low positive range of the assay. All samples from infected and vaccinated individuals had antibody levels above the cut-off (22 BAU/mL) providing an assay sensitivity of 100% (95% CI, 96.8% to 100.0%). However, two of the infected samples remained in the borderline area.

Among the 54 infected patients, Klinikum St. Georg Leipzig provided 36 serum samples with information on the severity of symptoms, which were assessed by WHO scores, with higher scores referring to more severe symptoms. Twenty-three samples were collected from mild symptom (WHO score = 2), four samples from medium symptom (WHO score = 3), and nine samples from heavy symptom patients (WHO score = 4). Despite the small number of medium and heavy symptom groups, the antibody levels rose with symptom severity (Figure 2B).

### 3.7. Interference and Cross-Reactivity

Substances such as hemoglobin, bilirubin, triglycerides, and heparin may interfere with the ELISA performance, leading to false positive or false negative results [26]. To evaluate their impact on our in-house assay, EDTA plasma samples with and without interfering substances were tested. Samples containing hemoglobin, bilirubin, triglycerides, or heparin showed no difference in antibody levels (CVs < 20%; typically < 14.8%) indicating no interference of these substances on test performance (Supplementary Figure S4).

Cross-reactivity of non-SARS-CoV-2-specific antibodies against the spike protein was evaluated using sera of patients with confirmed past infections with HIV 1/2, parvovirus B19, hepatitis A/B virus, cytomegalovirus, Epstein–Barr virus, herpes simplex virus, hCoV HKU-1, influenza H1N1, or influenza H5N1. The antibody levels of all 29 samples were below the cutoff (22 BAU/mL), indicating them as IgG-negative for anti-SARS-CoV-2 S protein (Supplementary Figure S4) and thereby showing no cross-reactivity.

### 3.8. Detection of IgG Antibodies in Different Sample Matrices

**Figure 3:**
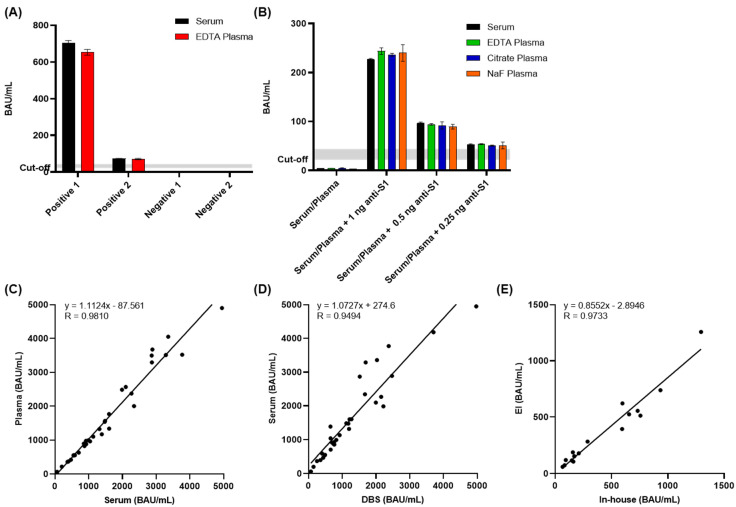
Equivalence of sample matrices. (**A**) Comparison of serum (black) and EDTA plasma (red) samples from two patients collected >14 days after symptom onset and two negative patients. (**B**) Serum (black), EDTA plasma (green), citrate plasma (blue), and NaF plasma (orange) were spiked with anti-S1 antibody. (**C**) Correlations between 32 positive serum and plasma samples collected from vaccinated donors (R = 0.9810). (**D**) Correlations between 32 positive serum and capillary blood DBS collected from vaccinated donors (R = 0.9494). (**E**) Correlations between in-house ELISA to EUROIMMUN quantitative ELISA using 16 whole-blood DBS samples collected from vaccinated donors (R = 0.9733).

The analysis of IgG antibodies against SARS-CoV-2 spike protein in different sample types has several advantages. First, interference or alteration of results by coagulation factors in serum or contaminating platelet and cellular elements of plasma will be excluded. Second, DBS samples are easier to collect (minimally invasive sample collection, smaller sample size, less patient fear of blood collection), less expensive, more stable during transport or storage, and able to be sampled remotely [27]. Importantly, serum and plasma samples collected from the same donors provided very similar anti-S IgG titers (Figure 3). The amount of anti-S antibodies present in serum and plasma samples from two patients collected >14 days after symptom onset as well as from two negative participants showed variations below 10% between serum and the corresponding plasma samples (Figure 3A). Additionally, serum and different plasma types (EDTA, citrate, and NaF) from one negative donor were spiked with 1, 0.5, 0.25, or 0 ng of anti-S1 antibody to mimic high, medium, and low positive samples as well as negative samples, respectively. None of the plasma samples differed significantly from the serum sample (Figure 3B). Furthermore, serum, plasma, and capillary blood DBS samples collected from 32 vaccinated donors (typically vaccinated three times) showed very good correlations of anti-S IgG titers between serum and plasma (Figure 3C) as well as good correlations between serum and DBS (Figure 3D). The in-house S-ELISA also showed a good correlation with a CE-labeled (European Economic Area) ELISA according to the manufacturer’s instructions (Euroimmun; Anti-SARS-CoV-2-QuantiVac-ELISA) for 16 whole blood pipetted DBS samples (Figure 3E). This suggests that the S-IgG ELISA is well-suited and can be adapted to different specimen types.

### 3.9. Time Course of Antibody Response during Vaccination

**Figure 4:**
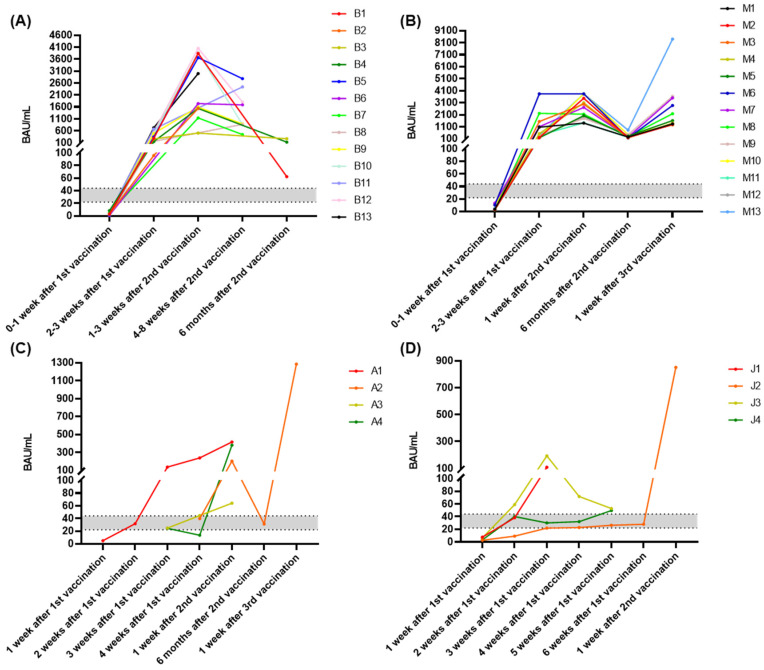
Time course of antibody titers (BAU/mL) determined following four different types of COVID-19 vaccination. Serum or DBS samples collected from donors vaccinated with (**A**) Pfizer-BioNTech (*n* = 13), (**B**) Moderna (*n* = 13), (**C**) AstraZeneca (*n* = 4), and (**D**) Johnson & Johnson (*n* = 4) vaccines. Blood samples were collected from five to seven time points for each vaccine before or after vaccination. Results were converted to binding antibody units (BAU/mL) using the absorbances recorded at 450 nm of six calibrators. The cutoff was 22 BAU/mL for the in-house ELISA. The grey zone above the cutoff (22–44 BAU/mL) indicates a borderline range.

The quantitative in-house ELISA allows monitoring the antibody response in individuals vaccinated with a COVID-19 vaccine. We evaluated anti-S-IgG antibody responses in 13 Pfizer-BioNTech, 13 Moderna, 4 AstraZeneca, and 4 Johnson & Johnson vaccine recipients (Figure 4A–D). None of the recipients were diagnosed with SARS-CoV-2. Serum or DBS samples were collected from 13 Pfizer-BioNTech vaccine recipients at five time points. The specific IgG antibodies were detected two to three weeks after the first vaccination with antibody levels ranging from 149.1 to 725.9 BAU/mL. Most Pfizer-BioNTech recipients reached the peak 1–3 weeks after second vaccination, with titers ranging from 1135.2 to 4048.6 BAU/mL, which decreased slightly 3–8 weeks after second vaccination.

For 13 Moderna vaccine recipients, generally higher IgG antibody levels ranging from 183.0 to 3840.0 BAU/mL were detectable from 2–3 weeks after the first vaccination than for Pfizer-BioNTech recipients. Recipients M6 and M8 had already reached the antibody peak at this time point, while the others reached the peak one week after the second vaccination. Six months after the second vaccination, antibody levels had decreased for all recipients, ranging from 156.8 to 817.3 BAU/mL. After a third vaccination (Moderna for M1–M8, BioNTech for M9, M10, and M13), all antibody levels increased again to high levels ranging from 1263.0 to 8402.8 (Figure 4B). For the four AstraZeneca vaccine recipients, DBS samples were collected at seven time points, however only one recipient had a relatively complete time course (A1). The antibodies were detected from two weeks after the first vaccination and increased until one week after the second vaccination. Two recipients (A3 and A4) provided DBS samples three weeks after the first vaccination, and their antibody levels remained negative and low positive until four weeks after the second vaccination, when the antibody levels started rising over the borderline. Similarly, Johnson & Johnson vaccine recipients produced relatively low anti-S titers (Figure 4D). Donors J2 and J4 remained in the very low positive antibody range. However, a third vaccination with another vaccine (Pfizer-BioNTech) in donors previously vaccinated with AstraZeneca (A2) and Johnson & Johnson (J2) triggered higher antibody levels (Figure 4C,D).

For most Pfizer-BioNTech and Moderna recipients, these data showed a similar response for anti-S-IgG antibodies, as generation of antibodies started two to three weeks after the first vaccination, peaked one week after the second vaccination, decreased afterwards, and returned to the peak after the third vaccination (received at least six months after the second vaccination). Although the study included only four AstraZeneca and four Johnson & Johnson vaccine recipients, it is still evident that the antibody levels were lower than in Pfizer-BioNTech and Moderna vaccine recipients.

### 3.10. Time Course of Antibody Response after Infection

**Figure 5:**
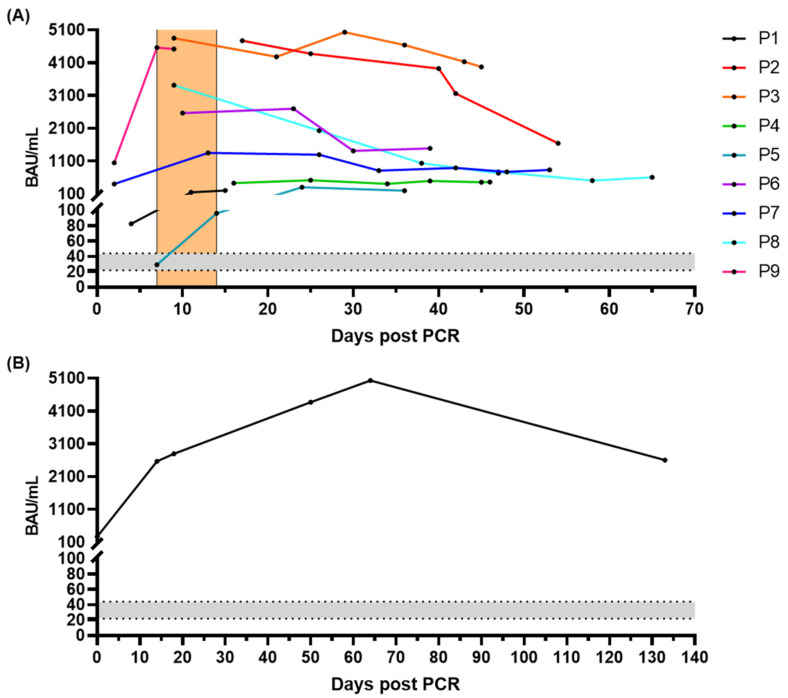
Time course of antibody titers (BAU/mL) during SARS-CoV-2 infection. (**A**) Serum samples collected from nine unvaccinated individual patients. The second week after PCR diagnosis is highlighted in orange. (**B**) DBS samples collected from a patient who was infected six months after the second vaccinations (Pfizer-BioNTech). The grey zone above the cutoff (22 BAU/mL) indicates the range of 22–44 BAU/mL, which is considered as positive.

The quantitative in-house ELISA also allowed monitoring of the antibody response in infected patients. For nine unvaccinated SARS-CoV-2-infected patients, follow-up samples were tested showing increasing antibody titers during the second week after PCR confirmation (Figure 5A; P1, P5, P7 and P9), and then remaining relatively stable for at least 53 days (Figure 5A; P3, P4, P5, and P7). The antibody levels of two patients decreased from days 9 and 17, but remained positive on days 54 and 65, respectively (Figure 5A; P2 and P8).

For one patient, who was infected six months after two vaccinations (Pfizer-BioNTech), the antibody titer remained increasing from 268.1 to 5033.3 BAU/mL over 64 days after symptoms onset and decreased to 2061.8 BAU/mL at day 133 (Figure 5B).

### 3.11. Stability

The stability of the in-house ELISA components was tested at 37 °C (accelerated). Controls and calibrators were assayed at a total of nine time points for the accelerated stability, showing an accelerated stability of >29 days (Supplementary Figure S5).

## 4. Discussion

In this study, we have established an iELISA to quantify IgG antibodies specifically recognizing the SARS-CoV-2 S protein. We optimized the ELISA conditions and assessed linearity, accuracy, precision analytical sensitivity, analytical specificity, and stability. From a clinical perspective, we assessed diagnostic sensitivity, specificity, cross-reactivity, and various sample matrices. The sensitivity for both infection and vaccination groups was 100% (95% CI, 96.8% to 100.0%) and the specificity was 98.3% (95% CI, 95.0% to 99.6%). No cross-reactivity was observed for several common viral infections. Collection of plasma or serum is currently the standard specimen for mainstream antibody testing in the marketplace. However, the cost, participant burden, and logistics associated with venipuncture blood collection are major barriers to community-based studies, especially in remote and impoverished areas where centrifuges or freezers may be limited. Therefore, the S-ELISA presented here was also validated for DBS, i.e., a drop of capillary blood collected from a finger prick. DBS samples have several advantages: minimally invasive sample collection and low cost; the requirements for sample processing are minimal [27]. Thus, they have already been used for several serological studies [28,29].

For the detection of SARS-CoV-2 infections, several serological assays have been reported based on different antigens and antibody combinations. A previous study validated five commercial SARS-CoV-2 IgG immunoassays on a cohort of recovered patients previously infected with SARS-CoV-2 (*n* = 363, 141–176 days after disease onset) and reported the lowest sensitivity of 53.71% for Abbott (anti-N), and the highest sensitivity of 93.11% for Roche (anti-N). The sensitivities of two anti-S1 tests from Euroimmun and Immundiagnostic were 77.13% and 89.23%, respectively, whereas the sensitivity of the anti-S1, S2 test from DiaSorin was 81.27%. The specificity of all five tests was above 99.0% [24]. Compared to these tests, our in-house S-protein-based ELISA has similar specificity, but a significantly higher sensitivity of 100% for samples collected at least 14 days after positive PCR test or symptoms onset. According to a previous study, the use of the trimeric S protein was associated with a higher sensitivity compared to the use of monomeric S and/or N proteins [30]. However, it should be noted that this 100% sensitivity might be related to a “lucky random” selection of sera. In our previous study, we reported an N-protein-based ELISA that showed 89.7% (61/68) sensitivity of samples collected at least 14 days after positive PCR test or symptoms onset [31]; the seven false negative samples all tested as positive in the S protein-based ELISA (Supplementary Figure S6A). Three pre-pandemic samples tested as false positive in the S-ELISA, one sample was from a rheumatoid arthritis patient, one from a non-small-cell lung carcinoma (NSCLC) patient, and the other from a lightly smoke woman. These three sera were correctly tested as negative in the N protein-based ELISA (Supplementary Figure S6B). Since the purity of the trimeric spike protein was 94% according to the manufacturer’s specifications, the false positive might be due to the epitopes recognized by antibodies being directed against other viral or HEK-293 cell proteins. These results further confirmed that S- and N-protein-based ELISA can be complementary to each other, thus improving the accuracy of the tests in screening infections.

In terms of monitoring the follow-up of IgG antibody titers against S protein in unvaccinated infected individuals, the S-ELISA showed similar downward trends to our previous N-ELISA [31]. Interestingly, a vaccinated (Pfizer-BioNTech) patient showed an upward trend in anti-S-IgG antibody level within 64 days after infection (Figure 5B), but was anti-N-IgG negative until day 64 (data not shown). Additionally, we tested seven more samples collected from vaccinated individuals who were infected in January/February 2022 either with the omicron or an undetermined variant of SARS-CoV-2. Among them, only one sample was positive in both S- and N-ELISA, whereas the other six samples were only anti-S-IgG positive (data not shown). This phenomenon has not been reported in other studies, and we speculate that this is due to the immune memory of mRNA vaccine recipients leading to a rapid memory antibody response upon exposure to the virus, resulting in the production of large amounts of anti-S-IgG and little anti-N-IgG. Thus, we assume that N-ELISA may not be sufficient to discriminate between infected and uninfected individuals among mRNA vaccine recipients. Combining the results of N- and S-ELISA might be important for future disease diagnosis and epidemiological studies.

Individual humoral responses to COVID-19 vaccination remain largely unpredictable. Recent studies in baseline anti-SARS-CoV-2 seronegative persons have shown that the immunogenicity of mRNA vaccination varies considerably between recipients [32,33]. Therefore, accurate serologic testing may help physicians to individualize dose adjustments or delay vaccination for patients based on actual anti-SARS-CoV-2 antibody levels. Different technologies and methods used for SARS-CoV-2 immunoassays detect different immunoglobulins for different viral epitopes, which may result in different detection limits, threshold values, and linear dynamic ranges [34]. An international standard has recently been introduced (i.e., the WHO standard, which enables the reporting of test results in binding antibody units per milliliter (BAU/mL)). A number of commercial tests are already using this set of standards. A previous study relying on three Pfizer-BioNTech vaccine recipients compared five commercial anti-SARS-CoV-2 antibodies immunoassays [35]. Roche, Beckman Coulter, and DiaSorin immunoassays can be converted to WHO binding antibodies units (BAU/mL), while SNIBE and Technogentics immunoassays were presented as arbitrary units per mL (AU/mL). Based on the observed good correlation and agreement of results between the five assays, Danese et al. concluded that the immunoassay test could be applied to detect anti-SARS-CoV-2 antibody neutralization responses in recipients of mRNA vaccination. In this study, our in-house ELISA was compared to the CE-labeled commercial Euroimmun Anti-SARS-CoV-2-QuantiVac-ELISA, providing similar results, i.e., recoveries from 89.3 to 102% on a series dilution of NIBSC standard, and good correlation (R = 0.9733) was achieved when both methods were used to analyze the same set of 16 DBS samples. The assay was validated with samples from 68 recipients of four vaccine types (Pfizer-BioNTech, Moderna, AstraZeneca, and Johnson & Johnson), all recipients showed a positive IgG result against the S protein. In a previous study, antibody responses to four SARS-CoV-2 vaccines were tested from high to low: Pfizer/BioNTech > AstraZeneca > Sputnik V > Sinopharm [36]. Our study including follow-up samples from 34 recipients indicated that the mRNA vaccines from Pfizer-BioNTech and Moderna provide a higher anti-S antibody level than the adenovirus-based vaccines from AstraZeneca and Johnson & Johnson, which is in line with recent immunogenicity studies [37,38]. Like other studies, we found that the immune response to vaccination is maintained for at least six months after two doses of the Pfizer-BioNTech and Moderna vaccines [39,40]; unfortunately, we were not able to draw precise conclusions about the persistence of immune responses to AstraZeneca and Johnson & Johnson vaccines due to the relatively low sample size.

## 5. Conclusions

We have shown the quantitative detection of anti-SARS-CoV-2 spike IgG antibodies to the trimeric S protein in an indirect ELISA with high sensitivity and specificity in serum, plasma, and DBS samples. This protocol is fast (<2 h) and requires less than 2 µL of serum or plasma, enabling even the analysis of DBS samples from the finger tips to simplify processing and reducing the costs. The in-house ELISA presented here allows for reliable and robust serological testing of past SARS-CoV-2 infections and COVID-19 vaccinations even for large-scale population testing strategies.

## Figures and Tables

**Table 1:** Diagnostic parameters of the indirect S-IgG ELISA.

Diagnostic Parameter	Value
Quantitation range	428.2 to 0.031 BAU/mL
LOD	0.114 BAU/mL
LOQ	0.345 BAU/mL
Precision	Inter-operator: 4.34% CV Inter-day: 7.83% CV
Reproducibility	4.00% CV
Cutoff	22 BAU/mL
Sensitivity	100% [95% CI, 96.8% to 100.0%]
Specificity	98.3% [95% CI, 95.0% to 99.6%]
Long-term stability	>29 d (37 °C)

Raw data of LOD, LOQ, and precision are listed in Tables S7 and S8. Long-term stability data are shown in Supplementary Figure S5.

## Data Availability

All data generated or analyzed during this study are included in this published article and its supplementary information files.

## Supplementary Materials

The following supporting information can be downloaded at: https://www.mdpi.com/article/10.3390/microorganisms10091812/s1, Table S1: Clinical parameters of vaccine recipients from Gemeinschaftspraxis Lipp/Amm/Lipp; Table S2: Clinical parameters of vaccine recipients from independent donors; Table S3: Clinical parameters of vaccine recipients from Klinikum St. Georg Leipzig; Table S4: Clinical parameters of infected patients from Klinikum St. Georg Leipzig; Table S5: Clinical parameters of infected patients from Krankenhaus Nordwest; Table S6: Clinical parameters of negative control samples; Table S7: Raw data of analytical sensitivity; Table S8: Raw data of precision and reproducibility; Figure S1: Optimization of the spike-protein ELISA; Figure S2: Linearity of the spike-protein ELISA; Figure S3: Interference; Figure S4: Cross-reactivity; Figure S5: Accelerate stability of spike-protein ELISA; Figure S6: Comparison of spike-protein and nucleocapsid-protein ELISA.
