# Supplementary material for: A Quantitative ELISA to Detect Anti-SARS-CoV-2 Spike IgG Antibodies in Infected Patients and Vaccinated Individuals
Source: Microorganisms. 2022 Sep 9;10(9):1812. doi: 10.3390/microorganisms10091812 (PMC9502828; doi:10.3390/microorganisms10091812)
Supplement: Supplementary file 1 [file microorganisms-10-01812-s001.zip › microorganisms-1902412-supplementary.pdf]

Table of Contents

Table S1: Clinical parameters of vaccine recipients from Gemeinschaftspraxis Lipp/Amm/Lipp **S2-S3**

Table S2: Clinical parameters of vaccine recipients from independent donors **S4-S8**

Table S3: Clinical parameters of vaccine recipients from Klinikum St. Georg Leipzig ... **S9-S10**

Table S4: Clinical parameters of infected patients from Klinikum St. Georg Leipzig ... **S11-S12**

Table S5: Clinical parameters of infected patients from Krankenhaus Nordwest **S13-S14**

Table S6: Clinical parameters of negative control samples........ **S15**

Table S7: Raw data of analytical sensitivity........ **S16-S17**

Table S8: Raw data of precision and reproducibility **S18-S19**

Figure S1: Optimization of the spike-protein ELISA **S20**

Figure S2: Linearity of the spike-protein ELISA **S21**

Figure S3: Interference........ **S22**

Figure S4: Cross-reactivity **S23**

Figure S5: Accelerate stability of spike-protein ELISA **S24**

Figure S6: Comparison of spike-protein and nucleocapsid-protein ELISA **S25**

Table S1. Clinical parameters of 46 serum or plasma samples obtained from vaccinated with mRNA (Pfizer-BioNTech) or adenovirus (Vaxzevira) vaccine at Gemeinschaftspraxis Lipp/Amm/Lipp (Leipzig, Germany) in the period from 14/01/2021 to 25/08/2021. Seventeen males and 29 females aged 20 to 81 years (mean age of 50 years) were included in the study.

Sample	Gender	Age	First vaccination	Second vaccination	Date of Blood was taken	Sample type
L1	w	43	14.01.2021	04.02.2021	22/07/2021	Serum
L2	w	44	14.01.2021	04.02.2021	22/07/2021	Serum
L3	m	35	14.01.2021	04.02.2021	21/07/2021	Serum
L4	w	42	15.01.2021	05.02.2021	23/07/2021	Serum
L5	w	42	21.01.2021	11.02.2021	22/07/2021	Serum
L6	w	54	15.01.2021	05.02.2021	22/07/2021	Serum
L7	w	63	14.01.2021	04.02.2021	22/07/2021	Serum
L8	w	62	14.01.2021	07.02.2021	22/07/2021	Serum
L9	w	28	23.01.2021	13.02.2021	22/07/2021	Serum
L10	w	26	11.02.2021	04.03.2021	23/07/2021	Serum
L11	m	52	04.05.2021 (Vaxzevria)	-	22/07/2021	Serum
L12	w	59	15.01.2021	06.02.2021	23/07/2021	Serum
L13	m	61	01.04.2021	24.06.2021	26/07/2021	Plasma
L14	m	65	23.01.2021	13.02.2021	25/07/2021	Plasma
L15	m	37	14.02.2021	07.03.2021	25/07/2021	Plasma
L16	m	57	12.05.2021	05.06.2021	25/07/2021	Plasma
L17	w	32	25.03.2021	21.04.2021	26/07/2021	Plasma
L18	m	32	14.02.2021	07.03.2021	25/07/2021	Plasma
L19	m	35	15.01.2021	05.02.2021	26/07/2021	Plasma
L20	w	41	05.05.2021	12.06.2021	25/07/2021	Plasma
L21	m	44	12.05.2021	05.06.2021	25/07/2021	Plasma
L22	w	39	15.03.2021	07.06.2021	26/07/2021	Plasma
L23	w	23	15.01.2021	05.02.2021	26/07/2021	Plasma
L24	w	52	03.05.2021	24.05.2021	25/07/2021	Plasma
L25	m	55	02.04.2021	14.07.2021	25/07/2021	Plasma
L26	w	45	23.02.2021	-	26/07/2021	Plasma
L27	w	50	14.01.2021	04.02.2021	29/07/2021	Plasma
L28	m	77	12.05.2021	05.06.2021	19/08/2021	Plasma
L29	w	74	12.05.2021	05.06.2021	19/08/2021	Plasma
L30	w	35	23.02.2021	16.03.2021	19/08/2021	Plasma
L31	w	60	15.01.2021	05.02.2021	19/08/2021	Plasma
L32	m	61	15.01.2021	05.02.2021	19/08/2021	Plasma
L33	w	75	29.03.2021	21.06.2021	19/08/2021	Plasma
L34	w	37	23.02.2021	16.03.2021	19/08/2021	Plasma
L35	w	20	13.02.2021	06.03.2021	19/08/2021	Plasma
L36	m	77	21.02.2021	01.02.2021	25/08/2021	Serum
L37	w	42	04.08.2021	25.08.2021	25/08/2021	Serum
L38	w	72	04.08.2021	25.08.2021	25/08/2021	Serum
L39	m	79	07.04.2021	30.06.2021	25/08/2021	Serum
L40	m	19	04.08.2021	25.08.2021	25/08/2021	Serum
L41	m	42	04.08.2021	25.08.2021	25/08/2021	Serum
L42	w	73	06.02.2021	27.02.2021	25/08/2021	Serum
L43	w	58	26.05.2021	16.06.2021	25/08/2021	Serum
L44	w	43	04.08.2021	25.08.2021	25/08/2021	Serum

Sample	Gender	Age	First vaccination	Second vaccination	Date of Blood was taken	Sample type
L45	m	65	29.04.2021	25.08.2021	25/08/2021	Serum
L46	w	81	21.02.2021	01.02.2021	25/08/2021	Serum

Table S2. Clinical parameters of 128 DBS samples obtained from 25 independent donors vaccinated with Moderna, Pfizer-BioNTech, AstraZeneca, and Johnson & Johnson vaccine in the period from 22/02/2021 to 17/12/2021. Thirteen males and eight females aged between 22 and 56 (mean age of 35 years) were included in the study.

Person	Gender	Age	First vaccination	Second vaccination	Third vaccination	Blood taken	Sample type
M1	m	28	15/05/2021 Moderna	12/06/2021 Moderna	12/12/2021 Moderna	14/05/2021	DBS
						29/05/2021	DBS
						19/06/2021	DBS
						12/12/2021	DBS
						19/12/2021	DBS
M2	m	32	17/05/2021 Moderna	14/06/2021 Moderna	02/12/2021 Moderna	17/05/2021	DBS
						31/05/2021	DBS
						21/06/2021	DBS
						14/12/2021	DBS
						14/12/2021	DBS
						17/12/2021	DBS
M3	w	25	19/05/2021 Moderna	16/06/2021 Moderna	17/12/2021 Moderna	17/05/2021	DBS
						02/06/2021	DBS
						23/06/2021	DBS
						16/12/2021	DBS
						31/12/2021	DBS
M4	m	32	19/05/2021 Moderna	16/06/2021 Moderna	17/12/2021 Moderna	17/05/2021	DBS
						02/06/2021	DBS
						23/06/2021	DBS
						16/12/2021	DBS
						31/12/2021	DBS
M5	m	36	24/05/2021 Moderna	21/06/2021 Moderna	14/12/2021 Moderna	17/05/2021	DBS
						07/06/2021	DBS
						28/06/2021	DBS
						14/12/2021	DBS
						03/01/2022	DBS
M6	m	24	07/05/2021 Moderna	04/06/2021 Moderna	04/12/2021 Moderna	07/05/2021	DBS
						22/05/2021	DBS

Person	Gender	Age	First vaccination	Second vaccination	Third vaccination	Blood taken	Sample type
						11/06/2021	DBS
						04/12/2021	DBS
						11/12/2021	DBS
						17/12/2021	DBS
M7	m	22	07/05/2021 Moderna	04/06/2021 Moderna	04/12/2021 Moderna	07/05/2021	DBS
						22/05/2021	DBS
						12/06/2021	DBS
						04/12/2021	DBS
						11/12/2021	DBS
						17/12/2021	DBS
M8	w	27	15/05/2021 Moderna	12/06/2021 Moderna	13/12/2021 Moderna	14/05/2021	DBS
						30/05/2021	DBS
						21/06/2021	DBS
						12/12/2021	DBS
						20/12/2021	DBS
						28/12/2021	DBS
M9	w	35	01/05/2021 Moderna	29/05/2021 Moderna	03/12/2021 BioNtech	07/05/2021	DBS
						14/05/2021	DBS
						21/05/2021	DBS
						04/06/2021	DBS
						29/11/2021	DBS
						10/12/2021	DBS
M10	m	31	11/05/2021 Moderna	08/06/2021 Moderna	03/12/2021 BioNtech	18/05/2021	DBS
						25/05/2021	DBS
						01/06/2021	DBS
						15/06/2021	DBS
						02/12/2021	DBS

Person	Gender	Age	First vaccination	Second vaccination	Third vaccination	Blood taken	Sample type
						10/12/2021	DBS
M11	w	22	11/05/2021 Moderna	08/06/2021 Moderna	-	18/05/2021	DBS
						25/05/2021	DBS
						01/06/2021	DBS
						15/06/2021	DBS
						09/12/2021	DBS
M12	w	32	11/05/2021 Moderna	08/06/2021 Moderna	-	18/05/2021	DBS
						25/05/2021	DBS
						01/06/2021	DBS
						15/06/2021	DBS
						08/12/2021	DBS
M13	w	22	17/05/2021 Moderna	14/06/2021 Moderna	03/12/2021 BioNtech	24/05/2021	DBS
						31/05/2021	DBS
						07/06/2021	DBS
						21/06/2021	DBS
						02/12/2021	DBS
						13/12/2021	DBS
A1	m	42	21/04/2021 Astra Zeneca	30/06/2021 Astra Zeneca	-	28/04/2021	DBS
						05/05/2021	DBS
						12/05/2021	DBS
						19/05/2021	DBS
						26/05/2021	DBS
						07/07/2021	DBS

Person	Gender	Age	First vaccination	Second vaccination	Third vaccination	Blood taken	Sample type
A2	m	69	22/02/2021 Astra Zeneca	13/05/2021 Astra Zeneca	03/12/2021 BioNtech	19/03/2021	DBS
						20/05/2021	DBS
						22/11/2021	DBS
						13/12/2021	DBS
A3	m	38	04/05/2021 Astra Zeneca	06/07/2021 Astra Zeneca	-	15/06/2021	DBS
						13/07/2021	DBS
A4	m	55	22/05/2021 Astra Zeneca	08/07/2021 BioNtech	-	12/06/2021	DBS
						03/07/2021	DBS
						15/07/2021	DBS
J1	m	55	25/06/2021 Johnson&Johnson	-	-	02/07/2021	DBS
						09/07/2021	DBS
						16/07/2021	DBS
						22/11/2021	DBS
J2	w	56	14/05/2021 Johnson&Johnson	15/08/2021 BioNtech	-	21/05/2021	DBS
						28/05/2021	DBS
						04/06/2021	DBS
						11/06/2021	DBS
						18/06/2021	DBS
						21/08/2021	DBS
J3	m	55	08/06/2021 Johnson&Johnson	-	-	15/06/2021	DBS
						22/06/2021	DBS
						29/06/2021	DBS
						06/07/2021	DBS
						13/07/2021	DBS
J4	w	57	08/06/2021 Johnson&Johnson	-		15/06/2021	DBS
						22/06/2021	DBS

Person	Gender	Age	First vaccination	Second vaccination	Third vaccination	Blood taken	Sample type
						29/06/2021	DBS
						06/07/2021	DBS
						13/07/2021	DBS
						08/12/2021	DBS
B1	w	22	04/05/2021 Pfizer-BioNTech	25/05/2021 Pfizer-BioNTech	-	11/05/2021	DBS
						18/05/2021	DBS
						24/05/2021	DBS
						01/06/2021	DBS
						25/11/2021	DBS
B2	m	32	16/03/2021 Pfizer-BioNTech	06/04/2021 Pfizer-BioNTech	-	19/03/2021	DBS
						23/03/2021	DBS
						29/03/2021	DBS
						16/03/2021	DBS
						06/04/2021	DBS
						21/04/2021	DBS
B3	w	33	01/05/2021 Pfizer-BioNTech	22/05/2021 Pfizer-BioNTech	-	08/05/2021	DBS
						15/05/2021	DBS
						21/05/2021	DBS
						22/11/2021	DBS
B4	w	39	03/05/2021 Pfizer-BioNTech	24/05/2021 Pfizer-BioNTech	-	12/05/2021	DBS
						17/05/2021	DBS
						23/05/2021	DBS
						01/06/2021	DBS
						24/11/2021	DBS

Table S3. Clinical parameters of 39 plasma samples obtained from nine independent donors vaccinated with mRNA (Pfizer-BioNTech) vaccine at the Klinikum St. Georg Leipzig in the period from 04/01/2021 to 03/02/2021. Four males and five females aged between 27 and 59 (mean age of 42 years) were included in the study.

Person	Age	Gender	First vaccination	Second vaccination	Blood taken	Sample type
B5	50	m	07/01/2021	28/01/2021	05/01/2021	Plasma
					27/01/2021	Plasma
					04/02/2021	Plasma
					09/03/2021	Plasma
B6	59	w	20/01/2021	03/02/2021	05/01/2021	Plasma
					10/02/2021	Plasma
					19/02/2021	Plasma
					12/04/2021	Plasma
B7	30	w	08/01/2021	27/01/2021	05/01/2021	Plasma
					28/01/2021	Plasma
					10/02/2021	Plasma
					23/02/2021	Plasma
					19/03/2021	Plasma
B8	55	m	04/01/2021	28/01/2021	n/a (before vaccination)	Plasma
					25/01/2021	Plasma
					25/02/2021	Plasma
B9	27	w	08/01/2021	29/01/2021	n/a (before vaccination)	Plasma
					28/01/2021	Plasma
					23/02/2021	Plasma
					11/03/2021	Plasma
B10	49	w	20/01/2021	03/02/2021	n/a (before vaccination)	Plasma
					10/02/2021	Plasma
					23/02/2021	Plasma
					23/02/2021	Plasma
					19/03/2021	Plasma
B11	35	m	04/01/2021	25/01/2021	n/a (before vaccination)	Plasma
					25/01/2021	Plasma
					22/03/2021	Plasma
B12	43	w	20/01/2021	03/02/2021	05/01/2021	Plasma
					10/02/2021	Plasma
					23/02/2021	Plasma
					17/03/2021	Plasma
B13	32	m	08/01/2021	29/01/2021	n/a (before vaccination)	Plasma
					15/01/2021	Plasma
					22/01/2021	Plasma

Person	Age	Gender	First vaccination	Second vaccination	Blood taken	Sample type
					26/01/2021	Plasma
					05/02/2021	Plasma
					10/02/2021	Plasma
					23/02/2021	Plasma

Table S4. Clinical parameters of 41 serum samples obtained from patients with confirmed SARS-CoV-2 infections and hospitalized at the Klinikum St. Georg Leipzig in the period from 02/04/2020 to 26/04/2020. Eighteen males and 21 females aged between 15 and 90 (mean age of 49 years) were included in the study. One sample of each patient was collected 23 to 51 days after PCR (mean of 30 days) and 23 to 55 days after symptom onset (mean of 40 days).

Sample	Gender	Age	Days after symptom onset	Days after PCR	WHO score
SG1	female	34	35	29	2
SG2	female	41	38	29	2
SG3	male	38	34	33	2
SG4	female	64	33	0	4
SG5	female	54	43	42	2
SG6	female	64	37	34	2
SG7	male	78	40	44	3
SG8	female	54	47	44	2
SG9	female	52	35	0	4
SG10	male	52	55	n/a	3
SG11	male	50	48	44	2
SG12	female	39	38	33	2
SG13	female	90	34	31	4
SG14	male	47	44	n/a	3
SG15	female	58	35	0	4
SG16	male	55	36	1	4
SG17	male	59	38	0	4
SG18	male	54	44	42	2
SG19	female	65	33	32	4
SG20	female	39	34	31	2
SG21	male	24	23	51	2
SG22	female	64	53	44	4
SG23	female	39	38	33	2
SG24	male	63	51	30	2
SG25	male	63	45	30	4
SG26	male	49	34	32	2
SG27	female	54	42	36	2
SG28	male	42	34	32	2
SG29	female	40	34	29	2
SG30	male	35	38	33	2
SG31	male	25	3	n/a	3
SG32	female	38	54	52	2
SG33	female	37	n/a	n/a	2
SG34	female	51	31	31	2
SG35	male	53	49	55	2
SG36	female	45	n/a	48	2
SG37	male	28	44	39	2
SG38	male	42	55	46	n/a

Sample	Gender	Age	Days after symptom onset	Days after PCR	WHO score
SG39	female	80	n/a	0	4
SG40	male	17	45	33	2
SG41	male	15	38	0	n/a

Table S5. Clinical parameters of 47 serum samples obtained from 9 patients aged between 42 and 97 (mean age of 69 years) with confirmed SARS-CoV-2 infections and hospitalized (except P5) at Krankenhaus Nordwest in Frankfurt in the period from 27/03/2020 to 02/06/2020. Samples were collected 0 to 65 days after PCR (mean of 29 days).

Patient	Age	Sample	Day after PCR
P1	97	F1	4
		F2	15
		F3	11
P2	81	F4	25
		F5	33
		F6	40
		F7	42
		F8	54
		F9	17
P3	76	F10	21
		F11	29
		F12	36
		F13	43
		F14	45
		F15	9
P4	59	F16	25
		F17	34
		F18	39
		F19	45
		F20	46
		F21	16
P5	n/a	F22	24
		F23	36
		F24	7
		F25	14
P6	83	F26	23
		F27	30
		F28	39
		F29	10
P7	62	F30	2
		F31	26
		F32	33
		F33	42
		F34	48
		F35	53
		F36	13
P8	42	F37	22
		F38	26
		F39	38

Patient	Age	Sample	Day after PCR
		F40	47
		F41	47
		F42	58
		F43	65
		F44	9
P9	62	F45	2
		F46	7
		F47	9

Table S6. Clinical parameters of 173 control serum samples collected from 2012 to 2018 aged from 34 to 79 (mean=63). Subgroups are partly overlapping.

**Prepandemic samples**	**173**
Rheumatoid arthritis	43
Ovarian cancer	2
Pancreas cancer	1
Colorectal cancer	1
Melanoma	2
Brain tumor	1
Neuroendocrine tumors (NETs)	1
Thymus cancer	1
Gastric carcinoma	2
NSCLC	2
male	54
female	36
Non-smokers:	39
Light-smokers:	36
heavy-smokers	17
Age	34-79 (Mean=63)

Table S7. Raw data of analytical sensitivity.

Analysis 1	Dilution factor	Replicate 1	Replicate 2	Replicate 3	Replicate 4	Replicate 5	Replicate 6	Replicate 7	Replicate 8	MW	SD	CV (%)
N1	16000	16.37	16.37	15.93	16.74	16.15	15.86	16.37	16.45	16.28	0.28	1.7%
N2	32000	8.17	8.45	7.82	8.73	8.24	7.82	8.31	8.24	8.22	0.30	3.7%
N3	64000	4.39	4.32	4.11	4.32	4.25	4.11	4.32	4.25	4.26	0.08	2.0%
N4	128000	2.22	2.42	2.15	2.22	2.36	2.09	2.29	2.22	2.25	0.11	4.8%
N5	256000	1.35	1.35	1.42	1.35	1.42	1.22	1.35	1.35	1.35	0.06	4.6%
N6	512000	0.82	0.95	0.82	0.88	0.88	0.82	0.88	0.82	0.86	0.05	5.4%
N7	1024000	0.55	0.62	0.55	0.55	0.62	0.55	0.55	0.62	0.57	0.03	5.7%
N8	2048000	0.42	0.55	0.42	0.48	0.42	0.48	0.48	0.48	0.47	0.04	9.1%
N9	4096000	0.35	0.42	0.35	0.35	0.42	0.42	0.42	0.35	0.38	0.03	8.6%
N10	8192000	0.42	0.48	0.42	0.48	0.55	0.48	0.48	0.48	0.47	0.04	7.5%

Analysis 2	Dilution factor	Replicate 1	Replicate 2	Replicate 3	Replicate 4	Replicate 5	Replicate 6	Replicate 7	Replicate 8	MW	SD	CV (%)
N1	16000	17.68	17.68	17.68	17.83	18.11	17.61	17.90	17.18	17.71	0.27	1.5%
N2	32000	9.41	9.41	9.41	9.27	9.34	9.68	9.75	9.20	9.43	0.19	2.0%
N3	64000	5.29	5.35	5.35	5.35	5.35	5.35	5.49	5.29	5.35	0.06	1.0%
N4	128000	3.16	3.36	3.43	3.30	3.43	3.36	3.36	3.23	3.33	0.07	2.0%
N5	256000	2.31	2.31	2.31	2.38	2.38	2.31	2.38	2.38	2.34	0.03	1.4%
N6	512000	1.72	1.85	1.85	1.79	1.79	1.85	1.79	1.79	1.80	0.03	1.8%
N7	1024000	1.52	1.59	1.59	1.52	1.52	1.59	1.59	1.52	1.56	0.03	2.1%
N8	2048000	1.39	1.46	1.46	1.46	1.52	1.46	1.46	1.39	1.45	0.03	2.4%
N9	4096000	1.33	1.46	1.39	1.46	1.46	1.39	1.46	1.39	1.42	0.03	2.3%
N10	8192000	1.39	1.52	1.59	1.72	1.59	1.59	1.59	1.59	1.57	0.06	3.5%

Analysis 3	Dilution factor	Replicate 1	Replicate 2	Replicate 3	Replicate 4	Replicate 5	Replicate 6	Replicate 7	Replicate 8	MW	SD	CV (%)
N1	16000	16.59	15.61	16.16	15.96	16.02	15.89	17.01	15.96	16.15	0.41	2.5%
N2	32000	8.65	8.38	8.58	8.38	8.45	8.32	8.58	8.05	8.42	0.17	2.0%

N3	64000	4.69	4.69	4.43	4.30	4.37	4.50	4.69	4.50	4.52	0.14	3.1%
N4	128000	2.55	2.68	2.55	2.68	2.62	2.68	2.68	2.55	2.63	0.06	2.2%
N5	256000	1.78	1.72	1.72	1.78	1.72	1.72	1.78	1.66	1.74	0.04	2.4%
N6	512000	1.21	1.34	1.27	1.27	1.27	1.27	1.27	1.15	1.26	0.05	4.2%
N7	1024000	0.95	1.02	1.02	0.95	1.02	0.95	1.02	0.95	0.99	0.03	3.2%
N8	2048000	0.76	0.89	0.83	0.89	0.89	0.83	0.95	0.83	0.86	0.04	5.2%
N9	4096000	0.76	0.76	0.83	0.76	0.89	0.76	0.89	0.70	0.79	0.07	8.4%
N10	8192000	0.76	0.83	0.83	0.89	0.89	0.83	0.89	0.76	0.83	0.04	5.4%

Table S8. Antibody titer (BAU/mL) of a positive pool analyzed in five different dilutions on different days, by different operators, and using different batches of the in-house ELISA to evaluate its precision and reproducibility.

Positive pool dilution	Person 1					Person 2					Person 3					Inter person		
	Replicate 1	Replicate 2	Replicate 3	Mean	**CV (%)**	Replicate 1	Replicate 2	Replicate 3	Mean	**CV (%)**	Replicate 1	Replicate 2	Replicate 3	Mean	**CV (%)**	Mean	SD	**CV (%)**
1:1000	379.0	383.4	381.9	381.4	**0.6**	389.2	409.6	415.7	404.8	3.4	414.8	413.7	415.4	414.6	0.2	400.3	17.1	4.3
1:2000	198.2	199.8	209.8	202.6	**3.1**	184.9	192.6	188.8	188.8	2.0	186.3	186.1	181.9	184.8	1.3	192.1	9.4	4.9
1:3000	139.1	141.7	133.6	138.1	**3.0**	126.8	129.7	125.7	127.4	1.6	119.5	123.2	124.0	122.2	2.0	129.3	8.1	6.3
1:5000	80.5	79.3	78.6	79.4	**1.2**	76.2	77.4	77.2	76.9	0.8	76.8	75.2	74.6	75.6	1.5	77.3	2.0	2.5
1:10000	42.2	43.9	43.3	43.1	**2.0**	40.5	41.6	41.6	41.2	1.4	39.2	41.0	40.0	40.1	2.2	41.5	1.6	3.8

Positive pool dilution	Day 1					Day 2					Day 3					Inter day		
	Replicate 1	Replicate 2	Replicate 3	Mean	**CV (%)**	Replicate 1	Replicate 2	Replicate 3	Mean	**CV (%)**	Replicate 1	Replicate 2	Replicate 3	Mean	**CV (%)**	Mean	SD	**CV (%)**
1:1000	379.0	383.4	381.9	381.4	**0.6**	357.0	338.6	366.4	354.0	4.0	373.2	395.0	396.4	388.2	3.4	374.6	18.1	4.8
1:2000	198.2	199.8	209.8	202.6	**3.1**	174.7	171.3	175.2	173.8	1.2	180.1	175.7	176.9	177.6	1.3	184.7	15.7	8.5
1:3000	139.1	141.7	133.6	138.1	**3.0**	148.0	133.9	129.8	137.2	6.9	116.4	116.6	118.6	117.2	1.1	130.9	11.8	9.1
1:5000	80.5	79.3	78.6	79.4	**1.2**	80.1	82.7	81.3	81.4	1.6	70.4	72.8	68.5	70.6	3.0	77.1	5.8	7.5
1:10000	42.2	43.9	43.3	43.1	**2.0**	43.3	44.2	41.7	43.1	2.9	36.3	36.6	36.6	36.5	0.5	40.9	3.8	9.3

Positive pool dilution	Lot 1					Lot 2					Lot 3					Inter Lot		
	Replicate 1	Replicate 2	Replicate 3	Mean	**CV (%)**	Replicate 1	Replicate 2	Replicate 3	Mean	**CV (%)**	Replicate 1	Replicate 2	Replicate 3	Mean	**CV (%)**	Mean	SD	**CV (%)**
1:1000	379.0	383.4	381.9	381.4	**0.6**	411.1	410.3	406.5	409.3	0.6	422.2	425.4	425.8	424.5	0.5	405.1	21.8	5.4
1:2000	198.2	199.8	209.8	202.6	**3.1**	189.5	189.2	201.5	193.4	3.6	193.1	197.1	204.6	198.3	2.9	198.1	4.6	2.3
1:3000	139.1	141.7	133.6	138.1	**3.0**	130.3	134.6	121.4	128.7	5.2	133.4	140.7	132.3	135.5	3.3	134.1	4.9	3.6

1:5000	80.5	79.3	78.6	79.4	**1.2**	72.3	74.4	76.5	74.4	2.9	81.7	79.5	77.6	79.6	2.6	77.8	3.0	3.8
1:10000	42.2	43.9	43.3	43.1	**2.0**	38.6	39.3	39.9	39.3	1.8	41.2	41.9	43.5	42.2	2.8	41.5	2.0	4.9

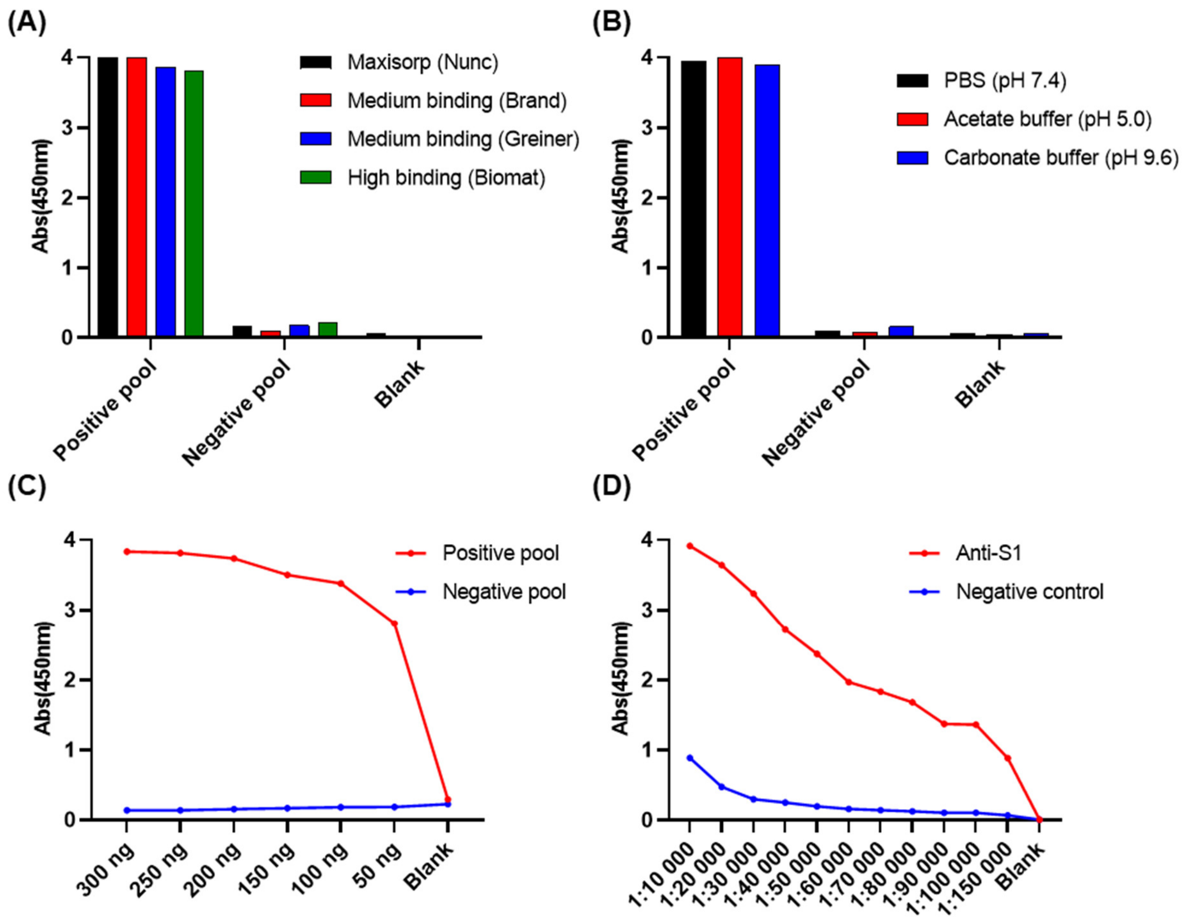

Figure S1. Optimization of the S-protein ELISA by testing different (A) microplate type (Maxisorp, Medium binding from Brand and Greiner, and High binding from Biomat), (B) coating buffer (PBS, acetate, and carbonate buffer), (C) antigen quantity (50-300 ng/well in PBS), and (D) dilution of anti-IgG secondary antibody (1:10,000 to 1:150,000 in Stabilzyme Select).

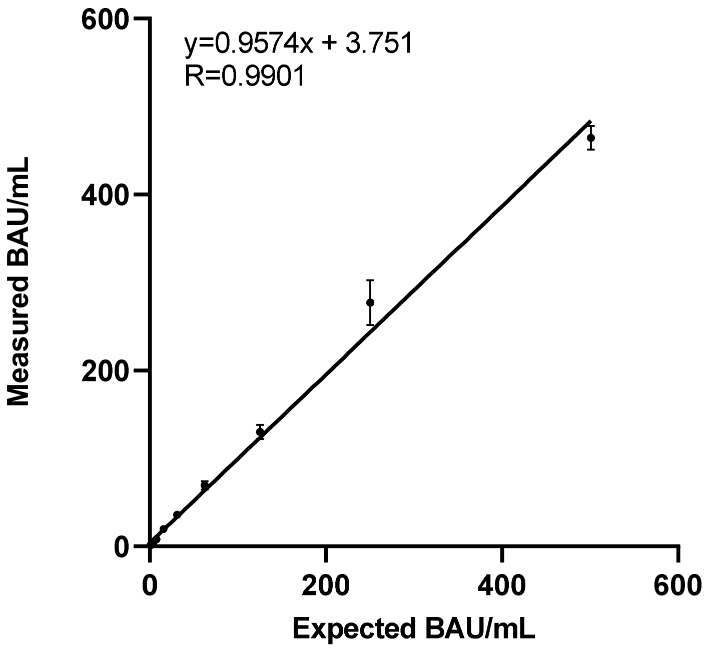

Figure S2. Linearity of the in-house SARS-CoV-2 S-IgG ELISA was determined by analyzing NIBSC 20/162 in a dilution series from 1:500 to 1:8,192,000. The eighteen dilutions of NIBSC 20/162 were assayed in eight replicates. Means of the measured values are plotted against the expected BAU/mL of the standard and standard deviations are shown as error bars.

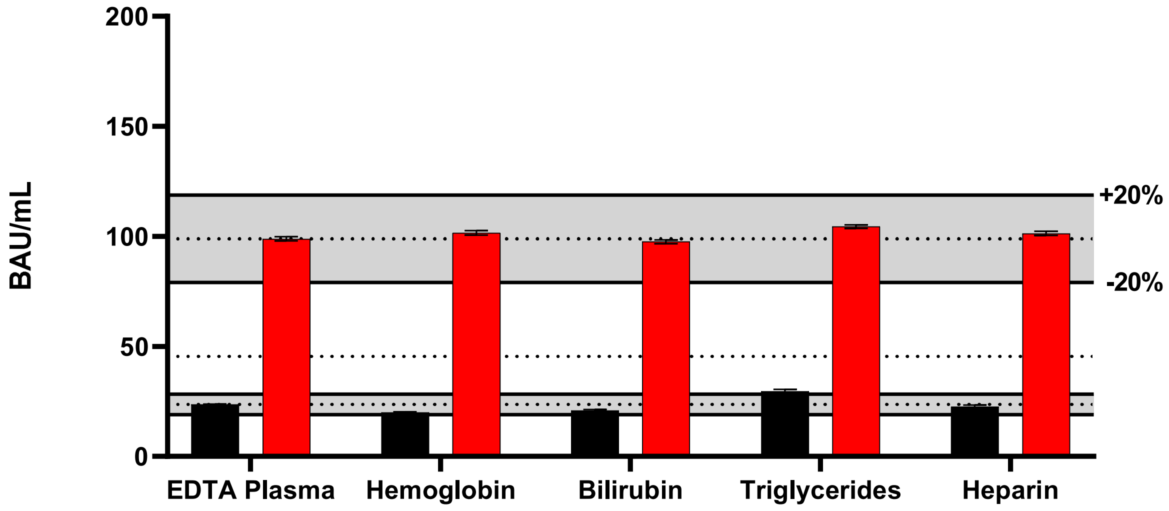

Figure S3. Influence of interfering substances: hemoglobin (~ 46 g/L), bilirubin (~ 0.35 g/L), triglycerides (~ 20 g/L), and heparin (~ 55 USP/mL) on the BAU of negative (black) and positive samples (red). Each sample was tested in triplicate in parallel. Dotted lines indicate average antibody level of the original plasma and the grey area indicates a relative standard deviation of ±20%.

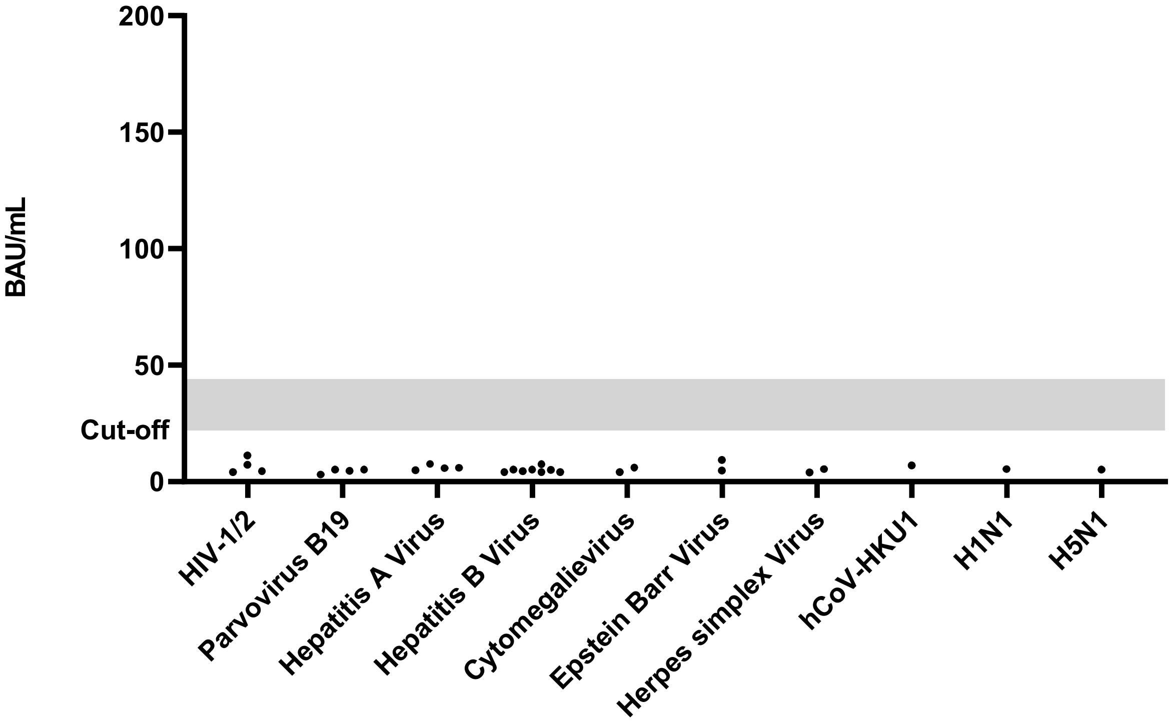

Figure S4. Antibody titers obtained by the in-house ELISA for serum samples tested positive for cytomegalievirus, herpes simplex virus, Epstein-Barr virus, HIV-1/2, parvovirus B19, hepatitis A/B virus, human coronavirus HKU1, and influenza H1N1 and H5N1. The grey zone above the cut-off (22 BAU/mL) indicates the range of 22-44 BAU/mL, which is considered as positive.

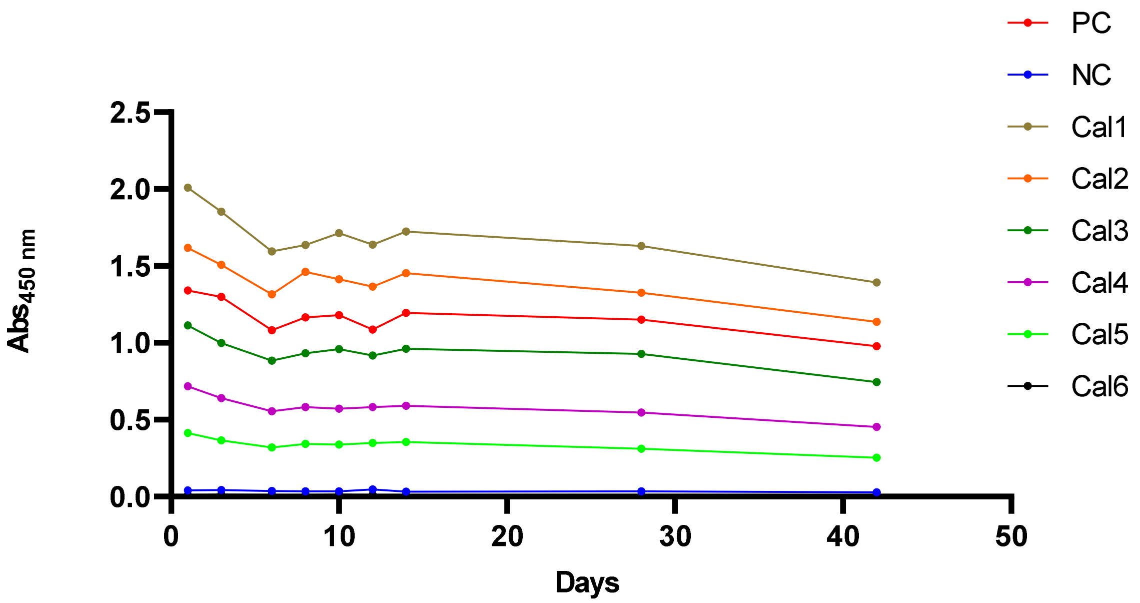

Figure S5. Trimeric Spike protein coated ELISA plates and all other assay reagents (negative control, calibrators, secondary antibody, substrates, and stop solution) were stored at 37 °C. Positive control was freshly prepared at each time point. Controls and calibrators were assayed at a total of ten time points.

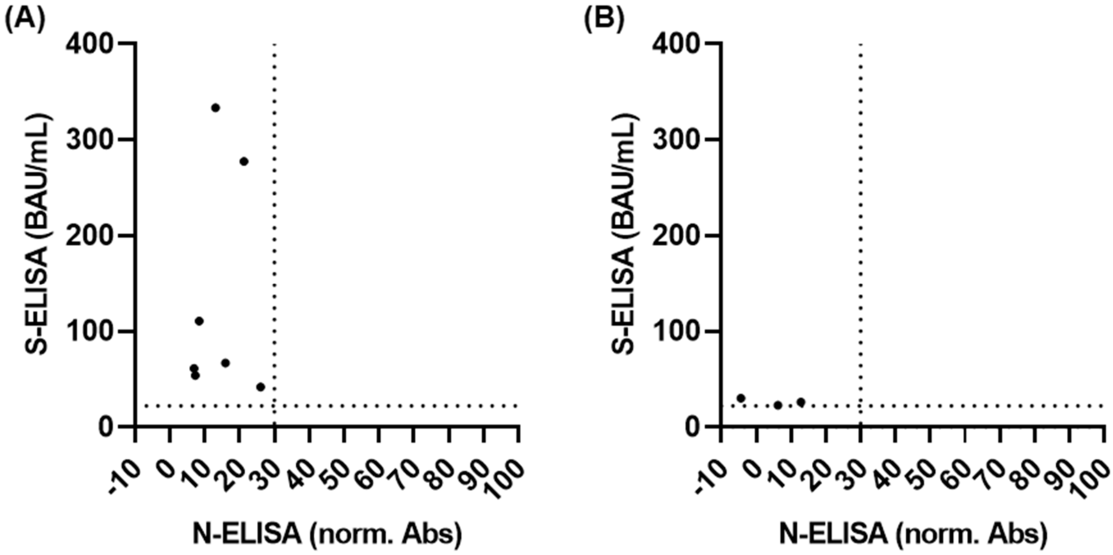

Figure S6. Serum samples incorrectly identified as positive and negative by the S- or N-protein based ELISA. (A) Seven samples collected more than 14 days after symptoms onset were tested as negative by N-ELISA. (B) Three samples detected as negative collected before 2018 in the N-ELISA (x-axis) were tested using the S-ELISA (y-axis). Cutoffs were 30% for the N-ELISA and 22 BAU/mL for the S-ELISA.
